# How embodied is cognition? fMRI and behavioral evidence for common neural resources underlying motor planning and mental rotation of bodily stimuli

**DOI:** 10.1093/cercor/bhad352

**Published:** 2023-10-06

**Authors:** Naz Doganci, Giannina Rita Iannotti, Sélim Yahia Coll, Radek Ptak

**Affiliations:** Laboratory of Cognitive Neurorehabilitation, Department of Clinical Neurosciences, Faculty of Medicine, University of Geneva, 1206 Geneva, Switzerland; Laboratory of Cognitive Neurorehabilitation, Department of Clinical Neurosciences, Faculty of Medicine, University of Geneva, 1206 Geneva, Switzerland; Department of Radiology and Medical Informatics, University Hospitals of Geneva, 1206 Geneva, Switzerland; Department of Neurosurgery, University Hospitals of Geneva, 1206 Geneva, Switzerland; Laboratory of Cognitive Neurorehabilitation, Department of Clinical Neurosciences, Faculty of Medicine, University of Geneva, 1206 Geneva, Switzerland; Department of Neurosurgery, University Hospitals of Geneva, 1206 Geneva, Switzerland; Division of Neurorehabilitation, University Hospitals of Geneva, 1206 Geneva, Switzerland; Laboratory of Cognitive Neurorehabilitation, Department of Clinical Neurosciences, Faculty of Medicine, University of Geneva, 1206 Geneva, Switzerland; Division of Neurorehabilitation, University Hospitals of Geneva, 1206 Geneva, Switzerland

**Keywords:** embodied cognition, fMRI, higher-order cognition, mental rotation, motor planning

## Abstract

Functional neuroimaging shows that dorsal frontoparietal regions exhibit conjoint activity during various motor and cognitive tasks. However, it is unclear whether these regions serve several, computationally independent functions, or underlie a motor “core process” that is reused to serve higher-order functions. We hypothesized that mental rotation capacity relies on a phylogenetically older motor process that is rooted within these areas. This hypothesis entails that neural and cognitive resources recruited during motor planning predict performance in seemingly unrelated mental rotation tasks. To test this hypothesis, we first identified brain regions associated with motor planning by measuring functional activations to internally-triggered vs externally-triggered finger presses in 30 healthy participants. Internally-triggered finger presses yielded significant activations in parietal, premotor, and occipitotemporal regions. We then asked participants to perform two mental rotation tasks outside the scanner, consisting of hands or letters as stimuli. Parietal and premotor activations were significant predictors of individual reaction times when mental rotation involved hands. We found no association between motor planning and performance in mental rotation of letters. Our results indicate that neural resources in parietal and premotor cortex recruited during motor planning also contribute to mental rotation of bodily stimuli, suggesting a common core component underlying both capacities.

## Introduction

Evolution imposes on brain structures constraints of energy-efficiency and cost-effectiveness, but also resistance to damage. Distributed information processing leads to highly redundant representations throughout brain networks, which appears to contradict principles of neural efficiency (Fair et al. 2009; Anderson 2010; Pessoa 2014). This limitation is counterbalanced by the potential of distributed systems to facilitate the emergence of new functions through the reuse and recombination of phylogenetically older sensorimotor processes (Anderson 2007). These processes act as essential building blocks of behavior that are supported by coalitions of multiple brain areas rather than by a single region (Pessoa 2023). While principles of neural reuse are difficult to demonstrate empirically, research in a particular domain suggests a crucial link between covert motor processes and visual–spatial transformations involved in mental rotation (Vingerhoets et al. 2002; Frick et al. 2009; Nishihara et al. 2015; Bennet and Reiner 2022; Menéndez Granda et al. 2022). At a first glance, motor planning and mental rotation appear to reflect entirely distinct cognitive capacities. For example, while the former requires the enactment of motor commands and the evaluation of action outcomes, the latter entails a mental transformation in the absence of physical execution. However, converging findings from behavioral, functional magnetic resonance imaging (fMRI), and electroencephalography (EEG) studies indicate that both tasks rely on internal representations and offline kinematics. For example, an almost linear relationship between rotational angle and reaction time (RT) supports the conclusion that mental rotation simulates physical transformations that would be necessary to actually rotate an object (Wohlschläger and Wohlschläger 1998). Psychophysical experiments reveal strong correlations between RTs in tasks measuring motor planning and mental rotation (Bennet and Reiner 2022), and show that conflicting motor plans interfere with mental rotation, thus suggesting the employment of overlapping cognitive components (Bach et al. 2014; Ptak et al. 2021). Motor planning and mental rotation are also positively correlated in children at 6 years old at the behavioral level (Toussaint et al. 2013). Along with other developmental studies, this suggests that acquired early sensorimotor capacities are applied in the context of higher-order cognition. The cognitive capacities enabling mental rotation in children thus emerge only once they have access to covert motor representations (Ornkloo and von Hofsten 2007; Frick et al. 2009; Jansen and Heil 2010).

A possible interpretation of these observations is that abstract kinematic plans produced by the motor system are reused during mental rotation (Ptak et al. 2017, 2021). This proposal is supported by neuroimaging results showing that motor planning and internally rehearsed actions rely on largely overlapping brain structures (Vingerhoets et al. 2002; Wraga et al. 2003; Hanakawa et al. 2008; Filimon 2010; Ptak et al. 2017; Cona and Scarpazza 2019). Thus, several studies observed similar activations of posterior parietal cortex (PPC) and premotor cortices when participants were engaged in movement planning and preparation (Beurze et al. 2007, 2010; Gallivan et al. 2011, 2013; Ariani et al. 2015; Pilacinski et al. 2018), motor imagery, or mental rotation (Zacks 2008; Milivojevic et al. 2009).

Despite these converging findings, the strength of the evidence suggesting a dependence of mental rotation on motor planning remains questionable. One important caveat is that cognitive and neural mechanisms underlying mental rotation are not the same for all stimulus types. In particular, several studies showed that mental rotation of bodily versus non-bodily stimuli differ regarding cognitive components, mental strategies and recruited brain regions (Corballis 1988; Sirigu and Duhamel 2001; Tomasino and Gremese 2016). For instance, while mental rotation of objects mainly activates bilateral parietal regions, mental rotation of hands additionally engages motor-related areas in premotor cortex (Kosslyn et al. 1998; Tomasino and Gremese 2016). This finding suggests the recruitment of covert motor mechanisms during the mental manipulation of body-related images, and hence the reliance of mental rotation on embodied representations (Kaltner et al. 2014; Iachini et al. 2019).

A major principle of neural reuse is that the cognitive architecture adapts to new tasks through a re-combination of basic neurocognitive elements (Anderson 2016). Several neuroimaging studies have reported overlapping activations when subjects planned motor actions or performed mental rotation. However, the overlap of activations in a specific brain area is not a sufficient argument for shared underlying functions. Activations may not necessarily reflect a causal relationship with hypothetical cognitive processes, or may even be entirely epiphenomenal to the studied function. More direct evidence for an interdependence would be obtained when activations associated with a purely motor task directly *predict* performance on an unrelated mental rotation task. To this aim, we used fMRI to identify brain regions that are activated when participants plan a simple finger movement. We then examined whether activations in these brain regions predicted performance on mental rotation tasks involving bodily or non-bodily stimuli that were performed outside the scanner. Based on the hypothesis that mental rotation reuses cognitive components of motor planning, we hypothesized that motor planning activity in parietal and premotor regions would directly predict mental rotation performance.

## Materials and methods

### Participants

For this study, 35 right-handed healthy participants participated to the fMRI part of this study. According to Hulley et al. (2013), we would need twenty-nine participants for a high power, based on our expectation of obtaining a correlation of *r =* 0.50 (here, between brain activations and mental rotation performance), with an effect size of 0.80 for *p =* 0.05. Due to technical problems including MRI trigger issues and excessive head movement, the data of four participants were excluded from the analyses. Another participant’s data had to be excluded due to his inability to follow the given instructions. Thus, data of thirty healthy participants with an age range of 21–43 years (mean = 27.4, SD = 5.7; 13 females) were considered for further fMRI analysis. Twenty-five out of these thirty participants performed the behavioral experiments outside of the MRI scanner on another day, and one participant was excluded as extreme outlier. Thus, the data of twenty-four participants (mean = 27.9 years, SD = 5.8; 10 females) were considered for further analysis on the behavioral part of this study.

Written informed consent was obtained prior to participating under a study procedure approved by the ethical commission of the Canton of Geneva (Switzerland) and following the standards set out by the Declaration of Helsinki. The inclusion criteria for this study were (i) right-handedness, (ii) absence of current or previous neurological or psychiatric disorders, and (iii) no incompatibilities with MRI scanning. Handedness of each participant was assessed using the Edinburgh Handedness Inventory (EHI; Oldfield 1971).

### Experimental designs

The study consisted of an fMRI and a behavioral session apart. The behavioral session outside the scanner examined participants’ mental rotation performance by considering two types of stimuli (hands and letters).

During fMRI, participants were required to perform simple finger movements, while alternating externally-triggered and participant’s selection of the action, as described in Doganci et al. (2023).

### MRI session

Functional and structural images were acquired using a 3 T Trio MRI scanner (Siemens Medical Solutions, Erlangen, Germany) with a 64-channel array coil. Whole-brain structural images were obtained with a high-resolution T1-weighted MPRAGE sequence (TR: 2,300 ms; TE: 1.96 ms; number of slices: 176; FA: 9°; voxel size = 1.0 mm isometric). Functional images consisted of a fast Echoplanar Imaging sequence (TR: 720 ms; TE: 30 ms; number of slices = 56 axial; voxel size = 2.5 mm isometric; flip angle (FA) = 50°). A field map for each participant was additionally acquired using the same field of view as the functional images.

**Fig. 1:**
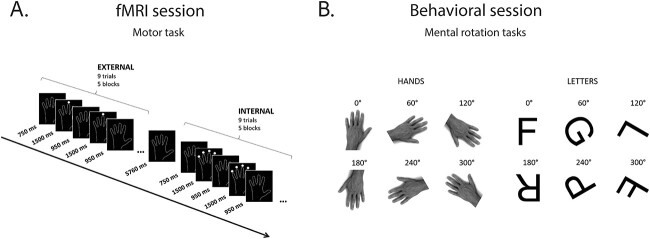
Example of a single run of the motor task (A) and demonstration of mental rotation tasks (B) with two types of stimuli: hands (left) and letters (right), each represented with six angular disparities.

The first step in investigating whether motor planning and mental rotation performance rely upon similar neural resources was to identify brain regions implicated in planning a simple finger movement. Motor planning consists of several steps (and associated sub-processes), including action (or effector) selection, initiation and control of the target action (Brass and Haggard 2008). Our fMRI experiment was intended to identify the selection step of motor planning. The fMRI experimental paradigm consisted of the image of a left or right hand outlined in white on black background (Fig. 1A). In the externally-triggered condition *(Ext)*, a white circle (cue) appeared randomly above one finger (except the thumb), while in the internally-triggered condition *(Int)*, four circles appeared simultaneously, one above each finger. Participants were asked to press down a button of a response box either with the cued finger (*Ext*) or with a randomly selected finger (*Int*). They were expected to provide a response within 1,500 milliseconds (ms) following the appearance of the cue, and responses exceeding this period were excluded from the analysis of RTs. The task was performed alternatively with the left or right hand in two distinct runs, separated by a short resting period. Hand order was counterbalanced across participants.

### Mental rotation session

All the stimuli were presented in random order on a 13.3″ laptop computer placed at a distance of 50 cm from the observer. The size of the hand stimuli at this distance was approximately 12.3° along their long axis and 9.2° along the short axis. The stimulus of the hand task was a black-and-white photograph of a neutral right male hand in palm-down view (Fig. 1B, left). Any marks on the hand that could potentially facilitate decisions were removed, and a left-hand version was created by mirror-imaging the right hand. The hand task consisted of 192 trials in four blocks of 48 trials, resulting in 32 trials per condition. The letter task consisted of 180 trials in three blocks of 60 trials, resulting in 30 trials per condition. The size of the letter stimuli at 50 cm distance was approximately 7.1° along their long axis and 8.4° along the short axis. For this task five capital letters (L, F, G, P, R) written in black Arial font were used in their canonical and a mirrored version (Fig. 1B, right). Five additional versions of each picture were obtained by rotating the picture plane around its perpendicular axis in 60° steps. The image set thus consisted of pictures of a left and right hand and each of the five letters presented at six different angular disparities (0°, 60°, 120°, 180°, 240°, 300°).

Since the hand task strongly relies on motor imagery (Vingerhoets et al. 2002), we registered vocal rather than manual responses to avoid motor interference with imagery of body parts (Cocksworth and Punt 2013). Hence, participants were asked to indicate whether pictures showed a left or right hand by saying “left” or “right,” or a letter in its canonical or mirror-reversed orientation by saying “correct” or “incorrect” as quickly and accurately as possible. In addition, participants were instructed not to look at their hands and to restrain any hand movement during the experiment. They were required to provide a response within 10 seconds upon appearance of the stimulus, after which the stimulus disappeared, and a new trial started.

### Statistical analysis

#### Imaging data analysis

Functional data, comprising *Ext* and *Int* conditions per participant, were preprocessed (slice-timing correction, realignment and unwarping using field maps, coregistration, spatial normalization, and smoothing) and analyzed using Statistical Parametric Mapping (SPM12; Wellcome Trust Centre for Neuroimaging, https://www.fil.ion.ucl.ac.uk/spm/software/spm12/). Scan-nulling regressors (Lemieux et al. 2007) and their Volterra expansion (Friston et al. 1998) for motion control in the MRI scanner as well as average time course from white matter and cerebrospinal fluid as additional nuisance regressors were included.

A two-factor GLM, accounting for left and right hand with *Int* and *Ext* conditions as regressors of interest, was performed for each participant at a first-level statistic, which was convolved by the hemodynamic response function. In order to identify brain areas that are crucial for motor planning, the *Int* and *Ext* conditions were contrasted irrespective of hand ((${R}_{Int}$+ ${L}_{Int}$)) > (${R}_{Ext}$+ ${L}_{Ext}$)). One-sample *t*-tests were performed at a group level, and activation results were visualized using the BrainNet Viewer toolbox (Xia et al. 2013).

#### Behavioral analysis of the mental rotation tasks

Vocal responses of each trial from individual participants were analyzed offline with a custom MATLAB script (please see Data availability section) to determine the onset of each response. Only RTs of correct responses (97.7% of all trials) with less than two standard deviations (SD) from the participants’ mean were considered for further analysis. The accuracy showed a ceiling effect (> 97% correct responses); therefore, it was not further analyzed.

In order to examine the main effects of stimulus (hands and letters) and angular disparity (0°, 60°, 120°, 180°, 240°, and 300°), as well as their interaction, chi-square difference tests were performed using the *phia* R package (R Core Team 2021, https://cran.r-project.org/web/packages/phia/index.html). The tests were adjusted for multiple comparisons with Bonferroni corrections. All RTs of correct trials were analyzed with linear mixed models (LMM) using the *lme4* R package (R Core Team 2021, https://cran.r-project.org/web/packages/lme4/index.html). Unlike analysis of variance, mixed models treat each trial individually as within-subject dependent measures, while the subject-level is treated as independent factor (Coll and Grandjean 2016). Importantly, this method is useful to control for within-subject variability, which was the random effect in RT analyses. In addition to main outcomes of our statistical tests, an effect size was calculated with the *MuMIn* R package (R Core Team 2021, https://cran.r-project.org/web/packages/MuMIn/index.html) for each statistical model that produced significant results, using a marginal *R*^2^ (*R*^2^_m_) reflecting the variance explained by fixed effects only, and a conditional *R*^2^ (*R*^2^_c_) reflecting variance explained by the whole model incorporating fixed and random effects (Nakagawa and Schielzeth 2013).

#### Relationship between fMRI task activation and mental rotation

We extracted maximum *T*-values for each participant and each significant cluster identified with the second-level fMRI analysis of the contrast (${R}_{Int}$+ ${L}_{Int}$) > (${R}_{Ext}$+ ${L}_{Ext}$). In order to correlate these *T*-values with a meaningful score that best reflects individual difficulty with MR, we computed normalized RT differences between the 180° (maximal rotation) and 0° condition (no rotation) for mental rotation of hands and letters as follows: (*RT 180°* – *RT 0°*)/(*RT 180° + RT 0°*). The greater the normalized difference, the longer a participant took to respond in the 180° condition compared to the 0° condition. According to Shapiro–Wilk normality test, the distribution of most activation values was non-normal, therefore we computed Spearman correlation coefficients in MATLAB between maximum *T*-values and normalized RT differences. We also correlated the maximum *T*-values with mental rotation RTs at 0° for control purposes, as no mental transformation should occur at this stage.

In order to investigate whether there was a hand effect, Spearman correlation coefficients were additionally computed between the maximum *T*-values from each cluster of left-hand performance (${L}_{Int}$ > ${L}_{Ext}$) and the mental rotation task performance of left-hand stimuli. However, one participant was excluded from this analysis due to her insufficient correct trials of mental rotation with left hand stimuli (*n* = 23). The same was performed for the right-hand run of the motor task with the right-hand stimuli of the mental rotation of hands (*n* = 24).

In addition to the correlation analyses, an LMM was performed using the *lme4* R package in order to investigate whether the motor planning regions together would overall predict mental rotation performance. In order to overcome multicollinearity and to reduce our variables for the LMM, first a principal component analysis (PCA) was performed, using the *Statistica* software (TIBCO Software Inc.). Once the variables were reduced, the principal component (PC) scores were extracted which were then set as fixed effects in the LMM. The other fixed effect was the stimulus type of the mental rotation tasks (hands and letters) and the dependent variable was the normalized RT difference (180° − 0°) of mental rotation of hands and letters, while controlling for within-subject variability which was set as the random effect. In the end, the model was constructed as the following: Normalized RT ~ PC1 * PC2 * stimulus type + (1 | Participant).

## Results

### Functional MRI results

**Fig. 2:**
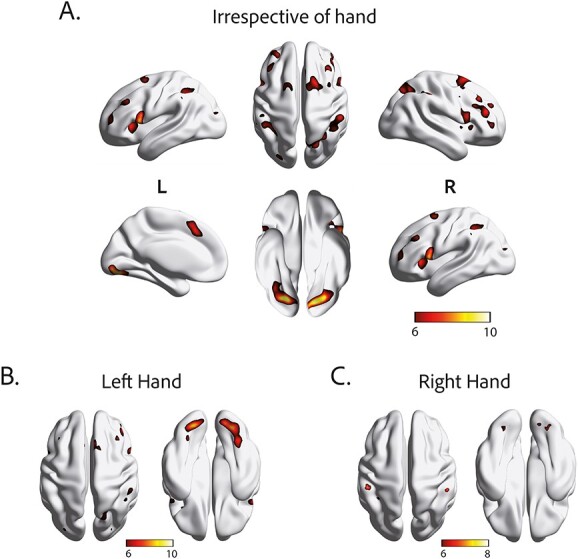
Significantly increased brain activations for the contrast of internally-triggered vs. externally-triggered condition of the motor task (A) irrespective of hand, and for performance of the left (B) and right hand (C). The colorbar indicates the *T*-values. The results are *FWE-corrected* with a threshold of *p < 0.05.* Modified from Doganci et al. (2023) with permission from Elsevier.

As shown in Fig. 2(A), the contrast of interest (${R}_{Int}$+ ${L}_{Int}$) > (${R}_{Ext}$+ ${L}_{Ext}$) yielded significant activations from sixteen clusters, which include the inferior parietal lobule (IPL) bilaterally, the right superior parietal lobule (SPL), premotor regions including the opercular part of left and right inferior frontal gyrus (IFG), left pre-SMA, and lateral and medial parts of the superior frontal gyrus (SFG). Interestingly, the highest activations were observed in the left lingual and right fusiform gyrus. Table 1 displays individual peaks, their corresponding MNI coordinates, cluster sizes and *T*-values.

**Table 1:** Significantly increased brain activations with their MNI coordinates, cluster sizes, and *T*-values of *Int > Ext* contrast irrespective of hand.

		MNI coordinates				
Structures	Side	x	y	z	Cluster size	*T*-value
Lingual gyrus	Left	−14	−82	−10	548	9.86
Fusiform gyrus	Right	24	−76	−10	524	9.45
Inferior frontal gyrus (pars opercularis)	Left	−50	12	10	232	8.64
Insula	Left	−32	26	4	82	7.38
Inferior parietal lobule	Right	56	−34	52	101	7.26
Medial superior frontal gyrus	Left	−4	22	44	276	7.25
Middle frontal gyrus	Right	40	38	18	69	7.2
Superior frontal gyrus	Right	20	10	64	126	7.12
Middle frontal gyrus	Left	−30	48	18	33	7.07
Middle frontal gyrus	Right	34	28	36	41	7.07
Inferior frontal gyrus (pars opercularis)	Right	56	14	14	156	7.02
Superior parietal lobule	Right	30	−64	52	193	6.91
Inferior parietal lobule	Left	−48	−46	50	86	6.73
Middle frontal gyrus	Right	38	42	4	17	6.68
Superior frontal gyrus	Left	−22	6	64	17	6.19

Note: Modified from Doganci et al. (2023) with permission from Elsevier. For the anatomical name of the regions, the AAL atlas was used (Tzourio-Mazoyer et al. 2002).

To further examine possible differences between hands, we analyzed the *Int > Ext* contrast for each hand separately. Left hand performance of the motor task resulted in increased activations in bilateral lingual gyrus, right SFG, bilateral IFG, right IPL, MFG, left middle occipital gyrus (MOG), as well as right precuneus (Fig. 2B, Supplementary Table S1). In contrast, right hand performance produced increased activations in bilateral IPL, left SPL, and bilateral fusiform gyrus (Fig. 2C, Supplementary Table S2). However, these increased activations were weaker and less diffuse compared to the left hand (Fig. 2B and C).

### Behavioral results of mental rotation tasks

**Fig. 3:**
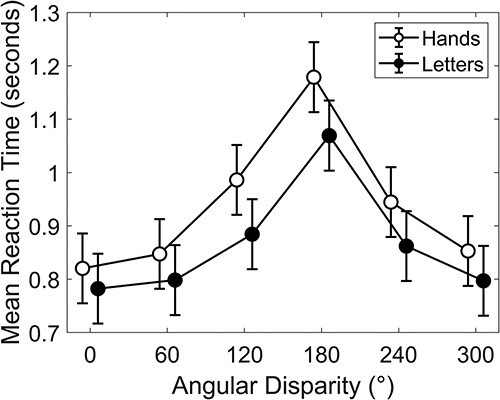
Mean RT in seconds as a function of angular disparity for mental rotation of hands and letters.

Figure 3 shows mean RTs in both tasks as a function of angular disparity. Both tasks yielded the expected increase of RT with increasing disparity, peaking at 180°. The LMM with stimulus and angular disparity as fixed effects, and subjects as random effect revealed significant effects of stimulus (*F*(1,8443) = 171.20, *p <* 0.001, *R^2^_m_ =* 0.012, *R^2^_c_ =* 0.243; for more details about the model comparison results, please see Supplementary Table S3) and angular disparity (*F*(5,8441) = 306.03, *p <* 0.001, *R^2^_m_ =* 0.115, *R^2^_c_ =* 0.35). Participants were slower for hands than letters, and slower for higher angular disparities. However, the interaction between both factors was also significant (*F*(5,8441) = 4.70, *p <* 0.001, *R^2^_m_ =* 0.129, *R^2^_c_ =* 0.368), indicating that angular disparity did not affect RT similarly for hands and letters. RTs were longer for hands than letters at all angular disparities (all *p <* 0.001), except for 0° and 60° (see Table 2). We further performed chi-square difference tests to examine differences between different angular disparities. These comparisons revealed significant differences between all disparities (all *P <* 0.001, for hands and letters), except for those that required only small rotations or departed for a similar amount from the 0° condition (0° vs. 60°; 0° vs. 300°; 60° vs. 300° and 120° vs. 240°; see Tables 3 and 4).

**Table 2:** Pairwise comparisons of stimulus in the mental rotation task for each angular disparity.

Degrees	Simple effect	*X* ^2^	df	*p_Bonferroni_*
0°	Hands vs. Letters	8.0033	1	0.028
60°	Hands vs. Letters	13.1219	1	0.002
120°	Hands vs. Letters	56.473	1	< 0.001
180°	Hands vs. Letters	61.6596	1	< 0.001
240°	Hands vs. Letters	37.3717	1	< 0.001
300°	Hands vs. Letters	17.1743	1	< 0.001

Note: *X*^2^: chi-square; *df*: degrees of freedom.

**Table 3:** Pairwise comparisons of all the angular disparities for mental rotation of hands.

Stimulus	Simple effect	*X* ^2^	df	*p_Bonferroni_*
Hands	0°–60°	4.178	1	1
Hands	0°–120°	155.8185	1	< 0.001
Hands	0°–180°	702.482	1	< 0.001
Hands	0°–240°	87.8609	1	< 0.001
Hands	0°–300°	6.0797	1	0.410
Hands	60°–120°	108.431	1	< 0.001
Hands	60°–180°	596.7915	1	< 0.001
Hands	60°–240°	53.454	1	< 0.001
Hands	60°–300°	0.1783	1	1
Hands	120°–180°	199.8406	1	< 0.001
Hands	120°–240°	9.6255	1	0.06
Hands	120°–300°	99.643	1	< 0.001
Hands	180°–240°	296.0179	1	< 0.001
Hands	180°–300°	575.6554	1	< 0.001
Hands	240°–300°	47.3742	1	< 0.001

Note: *X*^2^: chi-square; *df*: degrees of freedom.

**Table 4:** Pairwise comparisons of all the angular disparities for mental rotation of letters.

Stimulus	Simple effect	*X* ^2^	df	*p_Bonferroni_*
Letters	0°–60°	1.3797	1	1
Letters	0°–120°	55.9405	1	< 0.001
Letters	0°–180°	430.2036	1	< 0.001
Letters	0°–240°	34.2549	1	< 0.001
Letters	0°–300°	1.1686	1	1
Letters	60°–120°	39.4676	1	< 0.001
Letters	60°–180°	381.0604	1	< 0.001
Letters	60°–240°	21.7058	1	< 0.001
Letters	60°–300°	0.0093	1	1
Letters	120°–180°	178.0975	1	< 0.001
Letters	120°–240°	2.6733	1	1
Letters	120°–300°	40.9225	1	< 0.001
Letters	180°–240°	224.2111	1	< 0.001
Letters	180°–300°	386.8328	1	< 0.001
Letters	240°–300°	22.75	1	< 0.001

Note: *X*^2^: chi-square; *df*: degrees of freedom.

### Correlation analyses

To assess if the neural resources implicated during motor planning were engaged in mental rotation tasks, we evaluated the correlation between maximum *T*-values of the (${R}_{Int}$+ ${L}_{Int}$) > (${R}_{Ext}$+ ${L}_{Ext}$) contrast and performance in the mental rotation tasks. To obtain a simple index of RT performance, we computed for both mental rotation tasks normalized differences between RTs at 180° and 0°.

**Fig. 4:**
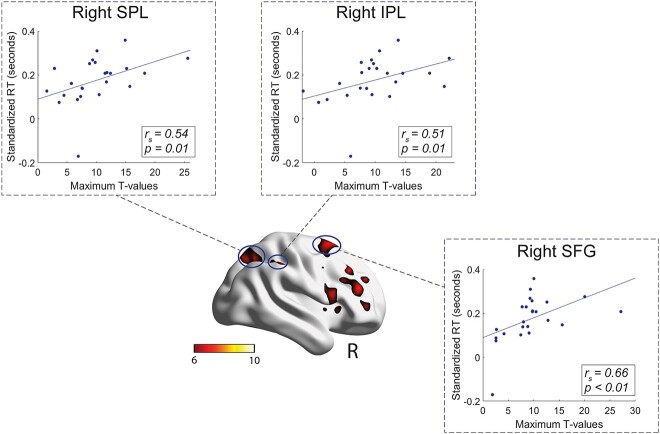
Plots of the strongest correlations between each participant’s maximum *T*-values in right SPL, right IPL, and right SFG from the motor task and normalized RT differences between 180° and 0° of the mental rotation task of hands.

For the hands task, fMRI activation in the right IPL, SPL and SFG significantly predicted mental rotation performance (Fig. 4), and less strong correlations were observed for the opercular part of the right IFG, the MFG and left pre-SMA (Table 5). This finding indicates that the more these regions were recruited during the motor task, the longer it took participants to answer in the hand mental rotation task. In contrast, no clusters showed a significant association with the normalized RT difference in the letter task (highest correlation: *r =* −0.31*, p =* 0.139). To further determine whether the significant associations predict a specific mental rotation process rather than general response speed, we also computed correlations between maximum *T*-values and simple RTs at 0°. These analyses did not identify any significant correlation for the hand task and the letter task (Table 5).

**Table 5:** The results of correlations between maximum *T*-values in each cluster resulted from the (${R}_{Int}$+ ${L}_{Int}$) > (${R}_{Ext}$+ ${L}_{Ext}$) contrast of the motor task and the normalized RT difference (180° − 0°) as well as the normalized RT of 0° of the mental rotation tasks of hands and letters.

		180° − 0°				0°			
		Hands		Letters		Hands		Letters	
Clusters	Side	*r_s_*	*p*	*r_s_*	*p*	*r_s_*	*p*	*r_s_*	*p*
Lingual gyrus	Left	0.19	0.385	0.02	0.911	0.04	0.866	−0.16	0.441
Fusiform gyrus	Right	0.26	0.211	−0.13	0.542	0.08	0.694	0.23	0.274
Inferior frontal gyrus (pars opercularis)	Left	0.27	0.208	0.05	0.802	0.15	0.494	0.19	0.360
Insula	Left	0.27	0.196	−0.05	0.802	0.05	0.821	0.24	0.252
Inferior parietal lobule	Right	0.51	0.011^a^	−0.13	0.545	0.14	0.526	0.11	0.595
Superior frontal gyrus (medial)	Left	0.48	0.017	−0.11	0.598	0.17	0.434	0.38	0.067
Middle frontal gyrus	Right	0.42	0.041	−0.25	0.244	0.28	0.186	0.3	0.149
Superior frontal gyrus	Right	0.66	< .001^a^	0.08	0.725	0.05	0.812	0.27	0.205
Middle frontal gyrus	Left	0.23	0.283	−0.03	0.879	0.12	0.586	−0.02	0.943
Middle frontal gyrus	Right	0.29	0.162	−0.14	0.518	0.15	0.486	0.24	0.256
Inferior frontal gyrus (pars opercularis)	Right	0.47	0.020	−0.05	0.818	0.06	0.768	0.07	0.734
Superior parietal lobule	Right	0.54	< .001^a^	0.06	0.771	0.21	0.329	0.33	0.113
Inferior parietal lobule	Left	0.17	0.431	−0.31	0.140	0.12	0.561	0.31	0.137
Middle frontal gyrus	Right	0.29	0.175	−0.27	0.208	0.05	0.831	−0.1	0.641
Middle frontal gyrus	Left	0.37	0.073	−0.25	0.244	0.31	0.147	0.37	0.076
Superior frontal gyrus	Left	0.26	0.266	0.17	0.439	−0.04	0.845	0.21	0.330

Note: *r_s_*: Spearman’s rank correlation coefficient.

^a^Strongest correlations.

Finally, to examine whether there was a similar relationship for distinct hands, we respectively correlated the maximum *T*-values of motor planning identified when participants used their left or right hand, with mental rotation performance implying pictures of the left or right hand. Among the left-hand clusters, activity in the right precuneus predicted mental rotation with left-hand stimuli, while the right SFG, the opercular part of the right IFG, IPL, MFG, and left MOG exhibited less strong correlations (Table 6). For mental rotation with right-hand stimuli, only the right fusiform gyrus positively correlated with performance. None of the activation clusters strongly predicted mental rotation performance with right-hand stimuli.

**Table 6:** The results of correlations between maximum *T*-values in each cluster resulted from the *Int > Ext* contrast of the left- and right-hand performances separately of the motor task and the normalized RT difference (180° − 0°) of the mental rotation of hand task with stimuli of left and right hand separately and respectively.

		Left hand				Right hand	
		180° − 0°				180° − 0°	
Clusters	Side	*r_s_*	*p*	Clusters	Side	*r_s_*	*p*
Lingual gyrus	Right	0.25	0.255	Inferior parietal lobule	Right	0.27	0.195
Lingual gyrus	Left	0.13	0.555	Inferior parietal lobule	Left	0.06	0.784
Superior frontal gyrus	Right	0.44	0.039	Inferior parietal lobule	Left	0.04	0.866
Inferior frontal gyrus (pars opercularis)	Right	0.44	0.038	Fusiform gyrus	Right	0.42	0.044
Inferior parietal lobule	Right	0.47	0.023	Fusiform gyrus	Left	0.18	0.398
Insula	Left	0.33	0.121				
Middle frontal gyrus	Right	0.46	0.029				
Middle occipital gyrus	Left	0.42	0.049				
Middle frontal gyrus	Right	0.39	0.07				
Precuneus	Right	0.52	0.012^a^				
Inferior frontal gyrus (pars opercularis)	Left	0.27	0.214				

Note: *r_s_*: Spearman’s rank correlation coefficient.

^a^Strongest correlations.

Among the right-hand clusters, only the right fusiform gyrus was correlated with right-hand stimuli (*r* = 0.42, *p* = 0.043), and the IPL predicted mental rotation with left-hand stimuli (*r* = 0.49, *p* = 0.018). These results indicate that activation associated with motor planning was a better predictor of behavioral performance when the motor task and the mental rotation task involved the same hand. In addition, more significant associations were found for the left (non-dominant) hand than the right hand.

### PCA and linear mixed model

**Fig. 5:**
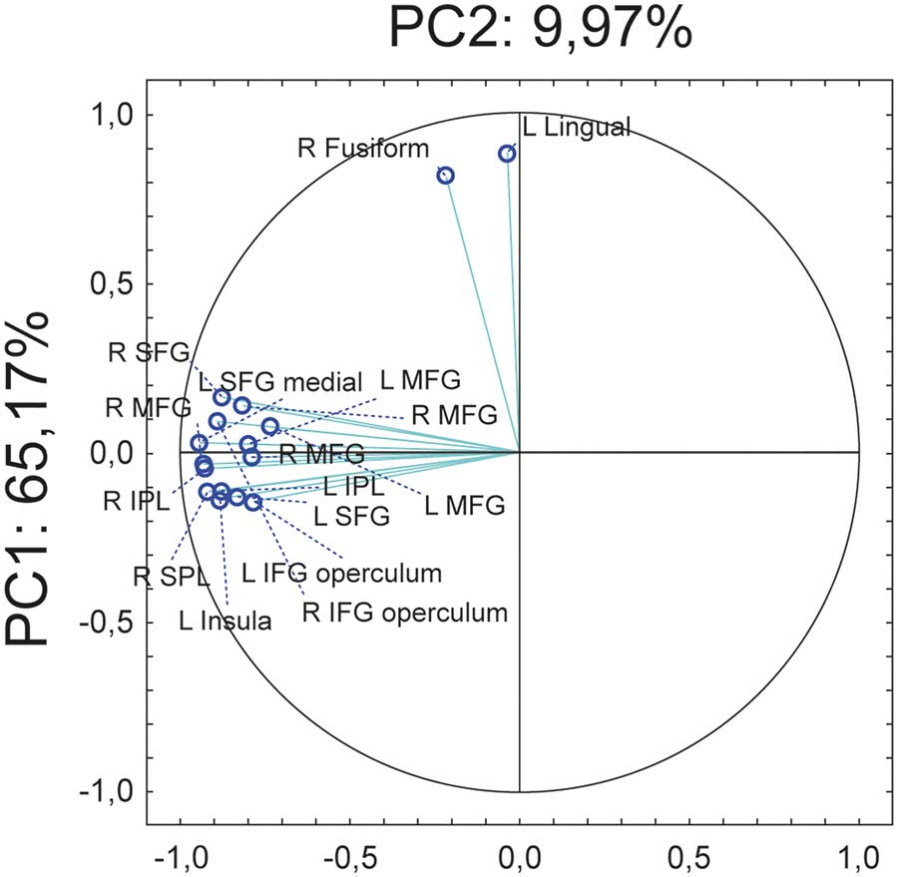
Plot of PCA results of maximum *T*-values from the 16 clusters, demonstrating the projection of the variables on the factor-plane (1 × 2). *L* stands for left and *R* for right.

Among the sixteen activation clusters identified with the (${R}_{Int}$+ ${L}_{Int}$) > (${R}_{Ext}$+ ${L}_{Ext}$) contrast in the fMRI results, most were located in frontoparietal areas, while two clusters occupied regions of inferior occipitotemporal cortex (Fig. 2A). Based on analyses of task-based functional connectivity, we previously showed that frontoparietal and occipitotemporal clusters are part of two segregated networks that have distinct contributions to intentional action (Doganci et al. 2023). The correlation analyses in the present study only examined individual clusters and did not consider possible functional subgroups that may predict mental rotation performance. We therefore performed a PCA on the maximum T-scores of all activation clusters to identify underlying large-scale components in the fMRI data. This analysis yielded two principal components (Fig. 5) that grouped together all frontoparietal activation clusters (component 1; 65.17% explained variance) and the two occipitotemporal clusters (component 2; 9.97% of explained variance).

**Fig. 6:**
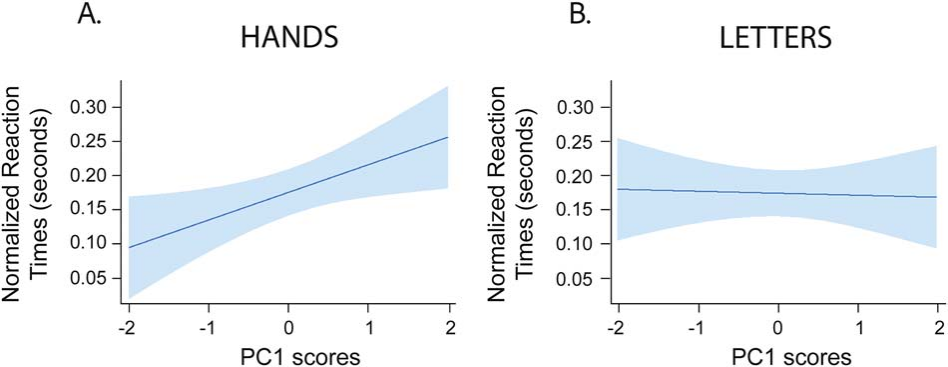
Linear mixed model interaction plot between the first principal component (PC1) scores for the motor task and stimulus type of mental rotation tasks.

We next examined whether component 1 and component 2 predicted mental rotation performance by incorporating them as fixed effects in an LMM, which treated the participant effect as a random variable. For further model comparison results, please refer to Supplementary Table S4. There were no significant main effects of component 1 (*F*(1,20) *=* 1.89*, p =* 0.184), component 2 (*F*(1,20) *=* 1.10*, p =* 0.307*)*, or stimulus type (*F*(1,20) *=* 0.004*, p =* 0.950*),* and no interaction between both components (*F*(1,20) *=* 0.589*, p=* 0.452*)* or all three factors (*F*(1,20) *=* 0.032*, p =* 0.860). While the interaction between component 2 and stimulus type did not reach significance (*F*(1,20) *=* 3.954*, p =* 0.061), we found a significant interaction between component 1 and stimulus type (*F*(1,20) *=* 5.315*, p =* 0.032*, R^2^_m_ =* 0.107, *R^2^_c_ =* 0.396). Simple effects analysis further revealed a significant effect for hands (*X*^2^(1) = 5.948, *p =* 0.015) but not for letters (*X*^2^(1) = 0.030, *p =* 0.860; Fig. 6). This finding confirms and extends our observations from the correlation analyses, by showing that frontoparietal functional activation related to motor planning predicts performance in a mental rotation task implying the mental processing of hand stimuli.

## Discussion

Our study shows that neural activation within frontoparietal areas involved in motor planning is a direct predictor of performance in mental rotation of bodily (hands), but not of non-bodily stimuli (letters). We identified motor planning areas by comparing activations to internally-triggered finger movements with externally-triggered ones (Doganci et al. 2023). As in previous studies, this comparison identified several areas of parietal and premotor cortex (Beudel and De Jong 2009; François-Brosseau et al. 2009), as well as inferior occipitotemporal regions. Since both movement conditions required simple finger movements, contrasting them excluded brain areas that are involved in motor execution (e.g. primary motor cortex). Active areas thus reflected components of the motor plan that are particularly important in the preparatory phases, such as the programming of kinematics, known to be associated with parietal and premotor activations.

Our correlation analyses identified several clusters in which activation was a positive predictor of mental rotation performance with hand, but not with letter stimuli. The highest correlations were found in the right PPC (SPL and IPL) and the right dorsal premotor cortex (PMd: SFG). A linear mixed model, testing the association between frontoparietal and occipitotemporal groups of clusters, showed that only the former was associated with mental rotation of hands. Taken together, our results support the interdependence between frontoparietal activations associated with motor planning and the capacity to mentally rotate bodily stimuli.

### Shared parietal and premotor resources between motor planning and mental rotation of hands

Previous studies have shown that frontoparietal regions play a predominant role in motor planning and mental transformations, particularly when bodily stimuli are involved (Gallivan et al. 2013; Ariani et al. 2015; Tomasino and Gremese 2016). However, in most previous studies, the link between motor planning and mental rotation was indirect, and they could not exclude the possibility that frontoparietal areas contributed to both functions independently. Our study shows that functional activations of the right IPL, SPL, and PMd measured with a motor task are more direct predictors of mental rotation of bodily stimuli. We observed exclusively positive correlations, indicating that longer RTs in mental rotation were associated with increased hemodynamic responses in the motor task. A similar pattern has been demonstrated in previous studies (Domagalik et al. 2014; Rao et al. 2014), suggesting that the recruitment of motor activity is related to increased effort in the rotation task.

The contribution of the PPC to mental rotation of hands fits with the role of this region in generating motor intentions, while being less implicated in motor execution (Desmurget et al. 2009). Damage to the PPC leads to impairments of motor imagery (Aflalo et al. 2015), a function that is often equated to mental rotation of bodily stimuli (Vingerhoets et al. 2002; Osuagwu and Vuckovic 2014). Within the PPC, the SPL is known to store visuospatial representations (Ratcliff 1979; Sack 2009) while the IPL is assumed to harbor motor representations (Pelgrims et al. 2009), both of which are essential building blocks for motor planning and mental rotation. Another region that is strongly engaged in covert action is the premotor cortex (Vingerhoets et al. 2002; Wraga et al. 2003; Zacks 2008). The dorsal part (PMd) in particular is activated during the preparation of goal-directed behaviors (Nakayama et al. 2022) and it also supports mental rotation processes by moderating visuospatial transformations (Sack et al. 2008; Cona et al. 2017). These findings indicate that mental rotation of hands is related to the generation of abstract kinematic plans within the IPL, SPL, and PMd.

### Mental rotation of hands relies predominantly on motor regions of the right hemisphere

We observed that brain regions predicting mental rotation of hands were mainly located in the right hemisphere. This seems to contradict several studies showing that mental rotation of bodily stimuli implicates the left hemisphere more than the right one, while the opposite is found for mental rotation of objects (Tomasino et al. 2004; Zacks 2008). However, the degree of lateralization appears to differ between anterior and posterior brain regions. For example, the right PPC plays a key role in spatial processing and visuospatial transformations irrespective of stimulus type (Corballis 1997; Formisano et al. 2002). Left lateralization to bodily stimuli is particularly observed for premotor cortex (Buiatti et al. 2011), which is partly in line with our results: though the best predictor of hand rotation was the right PMd, we also found a strong correlation in the left pre-SMA. Both regions are strongly activated when participants perform motor imagery of hands (Zhang et al. 2011; Simos et al. 2017), suggesting their potential implication in mental rotation of bodily stimuli. A possible cognitive explanation why we observed significant correlations mainly with right hemisphere regions is the application of a self-related strategy during mental rotation. Studies examining mechanisms of body ownership and self-related bodily processing identified right-lateralized frontoparietal areas as main correlates of limb movement perception and mental imagery of one’s own body (Naito et al. 2005; Blanke et al. 2010). When performing mental rotation of bodily stimuli participants often report attempting to match the stimulus with their own hand at different orientations (Parsons 1994). Such self-projection to bodily-related stimuli may therefore underlie the implication of right premotor regions in our study.

When conducting additional analyses separated for each hand, we found effects suggesting a strong association between the effector and the mental rotation target. Activity associated with motor planning implicating the left hand only predicted left-hand mental rotation in several right-hemisphere regions including the precuneus. Our data also showed that the correlation findings were mainly driven by fMRI data pertaining to the left hand. Several studies have implicated the precuneus in egocentric mental imagery and its dynamic updating (Cavanna and Trimble 2006; Weniger et al. 2009; Dordevic et al. 2022), which may specifically be required for mental rotation of hands. Moreover, Zapparoli et al. (2014) demonstrated that back views of hands engage the precuneus more than palm views, suggesting that the precuneus is involved in representations matching the canonical view of one’s own hand.

The correlation analyses thus show that functional activation associated with motor planning predicts mental transformations of hands if it implicates the same effector. This is in line with studies on clinical populations such as patients with hand dystonia (Fiorio et al. 2006), or upper limb amputees (Nico et al. 2004), who showed poorer performance for mental rotation with body parts representing the affected side. Together, these findings suggest that motor planning and the capacity to mentally rotate bodily stimuli rely on shared motor representations. Thus, our study substantiates the arguments that higher-level cognition is strongly embodied.

### From motor planning to mental rotation: neural reuse of embodied cognitive mechanisms

The correlation analyses considered brain activations in different clusters as independent predictors of mental rotation performance. However, functional task-evoked activations are themselves strongly correlated. A PCA across all activation clusters identified a dorsal (frontoparietal) group and a ventral (occipitotemporal) group of clusters representing distinct functional networks. Applying linear mixed modeling, we found that only activation of the frontoparietal group was a significant predictor of mental rotation. As in the correlation results, this activation only predicted hand mental rotation, while no significant effect was observed for letter stimuli. These findings indicate that mental rotation of bodily stimuli is strongly embodied, in that it relies on neural resources and mental processes that are recruited during motor planning (Ptak et al. 2017, 2021). However, motor planning is a complex function requiring the selection of target, movement, and associated effector, which raises the question whether all, or just some of these processes might be recruited for mental rotation. Since the fMRI clusters were identified by subtracting activations that were common to internally-triggered and externally-triggered action, we hypothesize that the associated process was close to the output side of motor planning, but before the specification of the effector. The rationale for this hypothesis is that activations associated with movement execution were identical in both conditions, and consequently activity of output regions such as the primary motor cortex was canceled. Modern models of motor planning integrate a copy of the motor command containing abstract kinematic representations that is sent as feedforward input and may serve as a prediction of the outcome of an intended action (Wong et al. 2015). Such a cognitive process is a good candidate for the reuse in other higher-order mental operations. It may therefore represent a core component that is implicated in motor planning and mental representations or transformations implying a body part.

Taken together, our study provides several arguments that motor planning and mental rotation share underlying neural resources, which is more direct than simply showing overlapping activations between the two tasks. We used two seemingly unrelated tasks that were performed on two different days. While the motor task focused on finger selection and execution of simple finger movements, the mental rotation tasks did not require any finger-related transformation nor did we ask our subjects to physically execute manual movements. This method goes beyond the simple measure of neural activations during two tasks and the emphasis on overlapping activations in specific cortical areas. Our approach provides more direct evidence that sensory and motor representations (such as kinematic plans) available during simple motor planning may be reused for an entirely different cognitive activity, together with the fundamentally implicated neural resources. A strong argument for neural reuse would be the presence of distinct networks whose alternative configurations are associated with different cognitive functions. This is what we found in the LMM analyses, as among two distinct networks observed during the motor planning task, only the frontoparietal network also contributed to mental rotation of bodily stimuli. Therefore, our findings support the main tenets of neural reuse, given that sub-parts of the dorsal network were recruited for a secondary function (D’Ambrosio and Colagè 2017). Nevertheless, causal evidence for neural reuse requires studies using techniques that allow causal inference, such as brain stimulation targeting the possible interactions between brain regions rather than the focus on isolated cortical areas (Pessoa 2023).

In conclusion, our results indicate that neural resources in parietal and premotor cortex recruited during motor planning also contribute to the mental representation, manipulation, and transformation of bodily stimuli. This finding suggests that a common core component underlying motor planning can be reused for a purely mental activity. While this core component does not explain mental rotation performance in its entirety, our results indicate that mental transformations are partly based on embodied mechanisms. We hypothesize that such neural reuse is a general principle of cognitive organization that may explain the emergence of higher-order processes based on low-level motor or sensory operations.

## Supplementary Material

Doganci_final_supplementary_material_bhad352

## Data Availability

The code for analyzing vocal responses of each trial from individual participants, as well as the data will be available upon request.

## References

[ref1] Aflalo T, Kellis S, Klaes C, Lee B, Shi Y, Pejsa K, Shanfield K, Hayes-Jackson S, Aisen M, Heck C, et al. Neurophysiology. Decoding motor imagery from the posterior parietal cortex of a tetraplegic human. Science. 2015:348(6237):906–910. 25999506 10.1126/science.aaa5417 PMC4896830

[ref2] Anderson ML . Massive redeployment, exaptation, and the functional integration of cognitive operations. Synthese. 2007:159(3):329–345.

[ref3] Anderson ML . Neural reuse: a fundamental organizational principle of the brain. Behav Brain Sci. 2010:33(4):245–266. 20964882 10.1017/S0140525X10000853

[ref4] Anderson ML . Précis of after phrenology: neural reuse and the interactive brain. Behav Brain Sci. 2016:39:e120. 26077688 10.1017/S0140525X15000631

[ref5] Ariani G, Wurm MF, Lingnau A. Decoding internally and externally driven movement plans. J Neurosci. 2015:35(42):14160–14171. 26490857 10.1523/JNEUROSCI.0596-15.2015 PMC6605426

[ref6] Bach P, Allami BK, Tucker M, Ellis R. Planning-related motor processes underlie mental practice and imitation learning. J Exp Psychol Gen. 2014:143(3):1277–1294. 24548280 10.1037/a0035604

[ref7] Bennet R, Reiner M. Shared mechanisms underlie mental imagery and motor planning. Sci Rep. 2022:12(1):2947. 35194088 10.1038/s41598-022-06800-9 PMC8863878

[ref8] Beudel M, De Jong BM. Overlap and segregation in predorsal premotor cortex activations related to free selection of self-referenced and target-based finger movements. Cereb Cortex. 2009:19(10):2361–2371. 19168663 10.1093/cercor/bhn254

[ref9] Beurze SM, de Lange FP, Toni I, Medendorp WP. Integration of target and effector information in the human brain during reach planning. J Neurophysiol. 2007:97(1):188–199. 16928798 10.1152/jn.00456.2006

[ref10] Beurze SM, Toni I, Pisella L, Medendorp WP. Reference frames for reach planning in human parietofrontal cortex. J Neurophysiol. 2010:104(3):1736–1745. 20660416 10.1152/jn.01044.2009

[ref11] Blanke O, Ionta S, Fornari E, Mohr C, Maeder P. Mental imagery for full and upper human bodies: common right hemisphere activations and distinct Extrastriate activations. Brain Topogr. 2010:23(3):321–332. 20333460 10.1007/s10548-010-0138-x

[ref12] Brass M, Haggard P. The what, when, whether model of intentional action. Neuroscientist. 2008:14(4):319–325. 18660462 10.1177/1073858408317417

[ref13] Buiatti T, Mussoni A, Toraldo A, Skrap M, Shallice T. Two qualitatively different impairments in making rotation operations. Cortex. 2011:47(2):166–179. 19914616 10.1016/j.cortex.2009.10.006

[ref14] Cavanna AE, Trimble MR. The precuneus: a review of its functional anatomy and behavioural correlates. Brain. 2006:129(3):564–583. 16399806 10.1093/brain/awl004

[ref15] Cocksworth RL, Punt TD. When the left hand does not know what the left hand is doing: response mode affects mental rotation of hands. Exp Brain Res. 2013:228(1):87–95. 23681291 10.1007/s00221-013-3540-2

[ref16] Coll SY, Grandjean D. Visuomotor integration of relevant and irrelevant angry and fearful facial expressions. Acta Psychol. 2016:170:226–238. 10.1016/j.actpsy.2016.09.001 27631573

[ref17] Cona G, Scarpazza C. Where is the “where” in the brain? A meta-analysis of neuroimaging studies on spatial cognition. Hum Brain Mapp. 2019:40(6):1867–1886. 30600568 10.1002/hbm.24496 PMC6865398

[ref18] Cona G, Panozzo G, Semenza C. The role of dorsal premotor cortex in mental rotation: a transcranial magnetic stimulation study. Brain Cogn. 2017:116:71–78. 28606388 10.1016/j.bandc.2017.06.002

[ref19] Corballis MC . Mental rotation and the right hemisphere. Brain Lang. 1997:57(1):100–121. 9126409 10.1006/brln.1997.1835

[ref20] Corballis MC . Recognition of disoriented shapes. Psychol Rev. 1988:95(1):115–123. 3281177 10.1037/0033-295x.95.1.115

[ref21] D’Ambrosio P, Colagè I. Extending epigenesis: from phenotypic plasticity to the bio-cultural feedback. Biol Philos. 2017:32(5):705–728.

[ref22] Desmurget M, Reilly KT, Richard N, Szathmari A, Mottolese C, Sirigu A. Movement intention after parietal cortex stimulation in humans. Science. 2009:324(5928):811–813. 19423830 10.1126/science.1169896

[ref23] Doganci N, Iannotti GR, Ptak R. Task-based functional connectivity identifies two segregated networks underlying intentional action. NeuroImage. 2023, 2023:268:119866. 36610680 10.1016/j.neuroimage.2023.119866

[ref24] Domagalik A, Beldzik E, Oginska H, Marek T, Fafrowicz M. Inconvenient correlation – RT–BOLD relationship for homogeneous and fast reactions. Neuroscience. 2014:278:211–221. 25158673 10.1016/j.neuroscience.2014.08.012

[ref25] Dordevic M, Hoelzer S, Russo A, García Alanis JC, Müller NG. The role of the precuneus in human spatial updating in a real environment setting—a cTBS study. Life. 2022:12(8):1239. 36013418 10.3390/life12081239 PMC9410530

[ref26] Fair DA, Cohen AL, Power JD, Dosenbach NUF, Church JA, Miezin FM, Schlaggar BL, Petersen SE. Functional brain networks develop from a “Local to Distributed” organization. PLoS Comput Biol. 2009:5(5):e1000381. 19412534 10.1371/journal.pcbi.1000381 PMC2671306

[ref27] Filimon F . Human cortical control of hand movements: parietofrontal networks for reaching, grasping, and pointing. Neuroscientist. 2010:16(4):388–407. 20817917 10.1177/1073858410375468

[ref28] Fiorio M, Tinazzi M, Aglioti SM. Selective impairment of hand mental rotation in patients with focal hand dystonia. Brain. 2006:129(1):47–54. 16150847 10.1093/brain/awh630

[ref29] Formisano E, Linden DEJ, Salle FD, Trojano L, Esposito F, Sack AT, Grossi D, Zanella FE, Goebel R. Tracking the Mind’s image in the brain I: time-resolved fMRI during visuospatial mental imagery. Neuron. 2002:35(1):185–194. 12123618 10.1016/s0896-6273(02)00747-x

[ref30] François-Brosseau FE, Martinu K, Strafella AP, Petrides M, Simard F, Monchi O. Basal ganglia and frontal involvement in self-generated and externally-triggered finger movements in the dominant and non-dominant hand. Eur J Neurosci. 2009:29(6):1277–1286. 19302163 10.1111/j.1460-9568.2009.06671.x

[ref31] Frick A, Daum MM, Walser S, Mast FW. Motor processes in Children’s mental rotation. J Cogn Dev. 2009:10(1–2):18–40.

[ref32] Friston KJ, Fletcher P, Josephs O, Holmes A, Rugg MD, Turner R. Event-related fMRI: characterizing differential responses. NeuroImage. 1998:7(1):30–40. 9500830 10.1006/nimg.1997.0306

[ref33] Gallivan JP, McLean DA, Smith FW, Culham JC. Decoding effector-dependent and effector-independent movement intentions from human parieto-frontal brain activity. J Neurosci. 2011:31(47):17149–17168. 22114283 10.1523/JNEUROSCI.1058-11.2011 PMC6623835

[ref34] Gallivan JP, McLean DA, Flanagan JR, Culham JC. Where one hand meets the other: limb-specific and action-dependent movement plans decoded from preparatory signals in single human frontoparietal brain areas. J Neurosci. 2013:33(5):1991–2008. 23365237 10.1523/JNEUROSCI.0541-12.2013 PMC6619126

[ref35] Hanakawa T, Dimyan MA, Hallett M. Motor planning, imagery, and execution in the distributed motor network: a time-course study with functional MRI. Cereb Cortex. 2008:18(12):2775–2788. 18359777 10.1093/cercor/bhn036 PMC2583155

[ref36] Hulley SB, Cummings SR, Browner WS, Grady DG, Newman TB. Total sample size required when using the correlation coefficient (r). Design Clin Res. 2013:218:79.

[ref37] Iachini T, Ruggiero G, Bartolo A, Rapuano M, Ruotolo F. The effect of body-related stimuli on mental rotation in children, young and elderly adults. Sci Rep. 2019:9(1):1169. 30718610 10.1038/s41598-018-37729-7 PMC6362092

[ref38] Jansen P, Heil M. The relation between motor development and mental rotation ability in 5- to 6-year-old children. Eur J Dev Sci. 2010:4(1):67–75.

[ref39] Kaltner S, Riecke BE, Jansen P. Embodied mental rotation: a special link between egocentric transformation and the bodily self. Front Psychol. 2014:5:505. 24917832 10.3389/fpsyg.2014.00505 PMC4042493

[ref40] Kosslyn SM, Digirolamo GJ, Thompson WL, Alpert NM. Mental rotation of objects versus hands: neural mechanisms revealed by positron emission tomography. Psychophysiology. 1998:35(2):151–161. 9529941

[ref41] Lemieux L, Salek-Haddadi A, Lund TE, Laufs H, Carmichael D. Modelling large motion events in fMRI studies of patients with epilepsy. Magn Reson Imaging. 2007:25(6):894–901. 17490845 10.1016/j.mri.2007.03.009

[ref42] Menéndez Granda M, Iannotti GR, Darqué A, Ptak R. Does mental rotation emulate motor processes? An electrophysiological study of objects and body parts. Front Hum Neurosci. 2022:16:983137. 36304589 10.3389/fnhum.2022.983137 PMC9592819

[ref43] Milivojevic B, Hamm JP, Corballis MC. Functional neuroanatomy of mental rotation. J Cogn Neurosci. 2009:21(5):945–959. 18702586 10.1162/jocn.2009.21085

[ref44] Naito E, Roland PE, Grefkes C, Choi HJ, Eickhoff S, Geyer S, Zilles K, Ehrsson HH. Dominance of the right hemisphere and role of area 2 in human kinesthesia. J Neurophysiol. 2005:93(2):1020–1034. 15385595 10.1152/jn.00637.2004

[ref45] Nakagawa S, Schielzeth H. A general and simple method for obtaining R2 from generalized linear mixed-effects models. Methods Ecol Evol. 2013:4(2):133–142.

[ref46] Nakayama Y, Sugawara SK, Fukunaga M, Hamano YH, Sadato N, Nishimura Y. The dorsal premotor cortex encodes the step-by-step planning processes for goal-directed motor behavior in humans. NeuroImage. 2022:256:119221. 35447355 10.1016/j.neuroimage.2022.119221

[ref47] Nico D, Daprati E, Rigal F, Parsons L, Sirigu A. Left and right hand recognition in upper limb amputees. Brain. 2004:127(1):120–132. 14607796 10.1093/brain/awh006

[ref48] Nishihara S, Imai F, Fujiki A, Majima Y. Interaction between mental rotation and manual rotation with and without motor planning. Psychology. 2015:6(9):1086–1095.

[ref49] Oldfield RC . The assessment and analysis of handedness: the Edinburgh inventory. Neuropsychologia. 1971:9(1):97–113. 5146491 10.1016/0028-3932(71)90067-4

[ref50] Ornkloo H, von Hofsten C. Fitting objects into holes: on the development of spatial cognition skills. Dev Psychol. 2007:43(2):404–416. 17352547 10.1037/0012-1649.43.2.404

[ref51] Osuagwu BA, Vuckovic A. Similarities between explicit and implicit motor imagery in mental rotation of hands: an EEG study. Neuropsychologia. 2014:65:197–210. 25446966 10.1016/j.neuropsychologia.2014.10.029

[ref52] Parsons LM . Temporal and kinematic properties of motor behavior reflected in mentally simulated action. J Exp Psychol Hum Percept Perform. 1994:20(4):709–730. 8083630 10.1037//0096-1523.20.4.709

[ref53] Pelgrims B, Andres M, Olivier E. Double dissociation between motor and visual imagery in the posterior parietal cortex. Cereb Cortex. 2009:19(10):2298–2307. 19168666 10.1093/cercor/bhn248

[ref54] Pessoa L . Understanding brain networks and brain organization. Phys Life Rev. 2014:11(3):400–435. 24819881 10.1016/j.plrev.2014.03.005 PMC4157099

[ref55] Pilacinski A, Wallscheid M, Lindner A. Human posterior parietal and dorsal premotor cortex encode the visual properties of an upcoming action. PLoS One. 2018:13(10):e0198051. 30300356 10.1371/journal.pone.0198051 PMC6177124

[ref56] Ptak R, Schnider A, Fellrath J. The dorsal frontoparietal network: a Core system for emulated action. Trends Cogn Sci. 2017:21(8):589–599. 28578977 10.1016/j.tics.2017.05.002

[ref57] Ptak R, Doganci N, Bourgeois A. From action to cognition: neural reuse, network theory and the emergence of higher cognitive functions. Brain Sci. 2021:11(12):1652. 34942954 10.3390/brainsci11121652 PMC8699577

[ref58] Rao NK, Motes MA, Rypma B. Investigating the neural bases for intra-subject cognitive efficiency changes using functional magnetic resonance imaging. Front Hum Neurosci. 2014:8:840. 25374527 10.3389/fnhum.2014.00840 PMC4204461

[ref59] Ratcliff G . Spatial thought, mental rotation and the right cerebral hemisphere. Neuropsychologia. 1979:17(1):49–54. 431809 10.1016/0028-3932(79)90021-6

[ref60] Sack AT . Parietal cortex and spatial cognition. Behav Brain Res. 2009:202(2):153–161. 19463696 10.1016/j.bbr.2009.03.012

[ref61] Sack AT, Jacobs C, Martino FD, Staeren N, Goebel R, Formisano E. Dynamic premotor-to-parietal interactions during spatial imagery. J Neurosci. 2008:28(34):8417–8429. 18716200 10.1523/JNEUROSCI.2656-08.2008 PMC6671055

[ref62] Simos PG, Kavroulakis E, Maris T, Papadaki E, Boursianis T, Kalaitzakis G, Savaki HE. Neural foundations of overt and covert actions. NeuroImage. 2017:152:482–496. 28323166 10.1016/j.neuroimage.2017.03.036

[ref63] Sirigu A, Duhamel JR. Motor and visual imagery as two complementary but neurally dissociable mental processes. J Cogn Neurosci. 2001:13(7):910–919. 11595094 10.1162/089892901753165827

[ref64] Pessoa L . The entangled brain. J Cogn Neurosci. 2023:35(3):349–360. 36007090 10.1162/jocn_a_01908 PMC11019915

[ref65] Tomasino B, Gremese M. Effects of stimulus type and strategy on mental rotation network: an activation likelihood estimation meta-analysis. Front Hum Neurosci. 2016:9:693. 26779003 10.3389/fnhum.2015.00693 PMC4704562

[ref66] Tomasino B, Vorano L, Skrap M, Gigli G, Rumiati RI. Effects of strategies on mental rotation performed by unilateral brain damaged patients. Cortex. 2004:40(1):197–199. 15174484 10.1016/s0010-9452(08)70949-3

[ref67] Toussaint L, Tahej P-K, Thibaut J-P, Possamai C-A, Badets A. On the link between action planning and motor imagery: a developmental study. Exp Brain Res. 2013:231(3):331–339. 24065290 10.1007/s00221-013-3698-7

[ref68] Tzourio-Mazoyer N, Landeau B, Papathanassiou D, Crivello F, Etard O, Delcroix N, Mazoyer B, Joliot M. Automated anatomical labeling of activations in SPM using a macroscopic anatomical parcellation of the MNI MRI single-subject brain. NeuroImage. 2002:15(1):273–289. 11771995 10.1006/nimg.2001.0978

[ref69] Vingerhoets G, De Lange FP, Vandemaele P, Deblaere K, Achten E. Motor imagery in mental rotation: an fMRI study. NeuroImage. 2002:17(3):1623–1633. 12414300 10.1006/nimg.2002.1290

[ref70] Weniger G, Ruhleder M, Wolf S, Lange C, Irle E. Egocentric memory impaired and allocentric memory intact as assessed by virtual reality in subjects with unilateral parietal cortex lesions. Neuropsychologia. 2009:47(1):59–69. 18789955 10.1016/j.neuropsychologia.2008.08.018

[ref71] Wohlschläger A, Wohlschläger A. Mental and manual rotation. J Exp Psychol Hum Percept Perform. 1998:24(2):397–412. 9606108 10.1037//0096-1523.24.2.397

[ref72] Wong AL, Haith AM, Krakauer JW. Motor planning. Neuroscientist. 2015:21(4):385–398. 24981338 10.1177/1073858414541484

[ref73] Wraga M, Thompson WL, Alpert NM, Kosslyn SM. Implicit transfer of motor strategies in mental rotation. Brain Cogn. 2003:52(2):135–143. 12821095 10.1016/s0278-2626(03)00033-2

[ref74] Xia M, Wang J, He Y. BrainNet viewer: a network visualization tool for human brain connectomics. PLoS One. 2013:8(7):e68910. 23861951 10.1371/journal.pone.0068910 PMC3701683

[ref75] Zacks JM . Neuroimaging studies of mental rotation: a meta-analysis and review. J Cogn Neurosci. 2008:20(1):1–19. 17919082 10.1162/jocn.2008.20013

[ref76] Zapparoli L, Invernizzi P, Gandola M, Berlingeri M, De Santis A, Zerbi A, Banfi G, Paulesu E. Like the back of the (right) hand? A new fMRI look on the hand laterality task. Exp Brain Res. 2014:232(12):3873–3895. 25150553 10.1007/s00221-014-4065-z

[ref77] Zhang H, Xu L, Wang S, Xie B, Guo J, Long Z, Yao L. Behavioral improvements and brain functional alterations by motor imagery training. Brain Res. 2011:1407:38–46. 21764038 10.1016/j.brainres.2011.06.038

