# Supplementary material for: How embodied is cognition? fMRI and behavioral evidence for common neural resources underlying motor planning and mental rotation of bodily stimuli
Source: Cereb Cortex. 2023 Oct 6;33(22):11146–56. doi: 10.1093/cercor/bhad352 (PMC10687356; doi:10.1093/cercor/bhad352)
Supplement: Doganci_final_supplementary_material_bhad352 [file doganci_final_supplementary_material_bhad352.docx]

**Supplementary material**

**Table S1.** Significantly increased brain activations with their MNI coordinates, cluster sizes, and T-values of *Int > Ext* contrast for the left-hand performance of the motor task.

|  |  | MNI coordinates | | |  |  |
| --- | --- | --- | --- | --- | --- | --- |
| Structures | Side | x | y | z | Cluster size | T-statistics |
| Lingual gyrus | Right | 22 | -78 | -10 | 508 | 9.57 |
| Lingual gyrus | Left | -20 | -80 | -8 | 361 | 8.89 |
| Superior frontal gyrus | Right | 18 | 12 | 64 | 333 | 7.28 |
| Inferior frontal gyrus (pars opercularis) | Right | 52 | 14 | 14 | 131 | 7.27 |
| Inferior parietal lobule | Right | 56 | -36 | 52 | 58 | 7.15 |
| Insula | Left | -32 | 26 | 4 | 26 | 7.03 |
| Middle frontal gyrus | Right | 42 | 40 | 22 | 60 | 7.02 |
| Middle occipital gyrus | Left | -32 | -80 | 16 | 20 | 6.99 |
| Middle frontal gyrus | Right | 36 | 30 | 38 | 31 | 6.97 |
| Precuneus | Right | 12 | -64 | 44 | 63 | 6.93 |
| Inferior frontal gyrus (pars opercularis) | Left | 50 | 14 | 10 | 27 | 6.59 |

**Table S2.** Significantly increased brain activations with their MNI coordinates, cluster sizes, and T-values of *Int > Ext* contrast for the right-hand performance of the motor task.

|  |  | MNI coordinates | | |  |  |
| --- | --- | --- | --- | --- | --- | --- |
| Structures | Side | x | y | z | Cluster size | T-statistics |
| Inferior parietal lobule | Right | 54 | -34 | 50 | 54 | 7.09 |
| Inferior parietal lobule | Left | -50 | -30 | 44 | 24 | 7 |
| Inferior parietal lobule | Left | -38 | -48 | 46 | 17 | 6.95 |
| Fusiform gyrus | Right | 26 | -76 | -8 | 56 | 6.93 |
| Fusiform gyrus | Left | -30 | -74 | -10 | 35 | 6.91 |

**Table S3.** Table of model comparison results for mental rotation behavioral performance.

| Test Effect | AIC | BIC | *X*^2^(df) | p-value |
| --- | --- | --- | --- | --- |
| Main Effect of Stimulus |  |  |  |  |
| *Model 1: Reaction time ~ Degrees + (1\|Subject)* | 1168.3 | 1224.7 |  |  |
| *Model 2: Reaction time ~ Stimulus + Degrees + (1\|Subject)* | 1003.2 | 1066.6 | 167.06(1) | < 0.0001 |
|  |  |  |  |  |
| Main Effect of Degrees |  |  |  |  |
| *Model 1: Reaction time ~ Stimulus + (1\|Subject)* | 2404 | 2432.2 |  |  |
| *Model 2: Reaction time ~ Stimulus + Degrees + (1\|Subject)* | 1003.2 | 1066.6 | 1410.7(5) | < 0.0001 |
|  |  |  |  |  |
| Interaction between *Stimulus* and *Degrees* |  |  |  |  |
| *Model 1: Reaction time ~ Stimulus + Degrees + (1\|Subject)* | 1003.23 | 1066.6 |  |  |
| *Model 2: Reaction time ~ Stimulus * Degrees + (1\|Subject)* | 989.74 | 1088.4 | 23.489(5) | 0.0003 |

Note: *AIC:* Akaike information criterion; *BIC:* Bayesian information criterion, *X*^2^: chi-square; *df:* degrees of freedom

**Table S4.** Table of model comparison results for mental rotation performance prediction by principle component scores resulting from motor planning task neural activations. PC1 and PC2 correspond to principal components 1 and 2 respectively.

| Test Effect | AIC | BIC | *X*^2^(df) | p-value |
| --- | --- | --- | --- | --- |
| Main effect of PC1 |  |  |  |  |
| *Model 1: Reaction time ~ PC2 + Stimulus + (1\|Subject)* | -97.331 | -87.975 |  |  |
| *Model 2: Reaction time ~ PC1 + PC2 + Stimulus + (1\|Subject)* | -97.319 | -86.092 | 1.9881(1) | 0.159 |
|  |  |  |  |  |
| Main effect of PC2 |  |  |  |  |
| *Model 1: Reaction time ~ PC1 + Stimulus + (1\|Subject)* | -97.342 | -87.986 |  |  |
| *Model 2: Reaction time ~ PC1 + PC2 + Stimulus + Degrees + (1\|Subject)* | -97.319 | -86.092 | 1.9775(1) | 0.16 |
|  |  |  |  |  |
| Main effect of Stimulus |  |  |  |  |
| *Model 1: Reaction time ~ PC1 + PC2 + (1\|Subject)* | -99.316 | -89.96 |  |  |
| *Model 2: Reaction time ~ PC1 + PC2 + Stimulus + (1\|Subject)* | -97.319 | -86.092 | 0.0033(1) | 0.95 |
|  |  |  |  |  |
| Interaction between PC1 and PC2 |  |  |  |  |
| *Model 1: Reaction time ~ PC1 + PC2 + Stimulus + (1\|Subject)* | -97.319 | -86.092 |  |  |
| *Model 2: Reaction time ~ PC1 + PC2 + Stimulus + PC1:PC2 + (1\|Subject)* | -96.016 | -82.918 | 0.6968(1) | 0.404 |
|  |  |  |  |  |
| Interaction between PC1 and Stimulus |  |  |  |  |
| *Model 1: Reaction time ~ PC1 + PC2 + Stimulus + (1\|Subject)* | -97.319 | -86.092 |  |  |
| *Model 2: Reaction time ~ PC1 + PC2 + Stimulus + PC1:Stimulus + (1\|Subject)* | -100.147 | -87.049 | 4.8281(1) | 0.028 |
|  |  |  |  |  |
| Interaction between PC2 and Stimulus |  |  |  |  |
| *Model 1: Reaction time ~ PC1 + PC2 + Stimulus + (1\|Subject)* | -97.319 | -86.092 |  |  |
| *Model 2: Reaction time ~ PC1 + PC2 + Stimulus + PC2:Stimulus + (1\|Subject)* | -98.909 | -85.81 | 3.5895(1) | 0.058 |
|  |  |  |  |  |
| Interaction between PC1, PC2 and Stimulus |  |  |  |  |
| *Model 1: Reaction time ~ (PC1 + PC2 + Stimulus)^2 + (1\|Subject)* | -101.31 | -84.472 |  |  |
| *Model 2: Reaction time ~ PC1 * PC2 * Stimulus + (1\|Subject)* | -99.35 | -80.638 | 0.038(1) | 0.845 |

Note: *AIC:* Akaike information criterion; *BIC:* Bayesian information criterion, *X*^2^: chi-square; *df:* degrees of freedom
